# Age and sex specific target of blood pressure for the prevention of cardiovascular event among the treatment naive hypertensive patients

**DOI:** 10.1038/s41598-020-78641-3

**Published:** 2020-12-09

**Authors:** Hyoungnae Kim, Seulbi Lee, Eunhee Ha, Soon Hyo Kwon, Jin Seok Jeon, Hyunjin Noh, Dong Cheol Han, Hyung Jung Oh, Dong-Ryeol Ryu

**Affiliations:** 1 Department of Internal Medicine, Soonchunhyang University Seoul Hospital, Seoul, Republic of Korea; 2 Department of Medical Science, School of Medicine, Ewha Womans University, Seoul, Republic of Korea; 3 Department of Occupational and Environmental Medicine, School of Medicine, Ewha Womans University, Seoul, Republic of Korea; 4 Research Institute for Human Health Information, Ewha Womans University Mokdong Hospital, Seoul, Republic of Korea; 5 Department of Nephrology, Sheikh Khalifa Specialty Hospital, Al Shohadaa Road, Exit 119, Ras Al Khaimah, UAE; 6 Department of Internal Medicine, School of Medicine, Ewha Womans University, Gonghang-daero 260, Gangseo-gu, Seoul, 07804 Republic of Korea

**Keywords:** Cardiology, Cardiovascular diseases

## Abstract

The time at which hypertension treatment should be initiated for different age groups and sexes remains controversial. We aimed to determine whether the association between blood pressure (BP) and major adverse cardiovascular events (MACE) varies with age and sex. This study enrolled 327,328 subjects who had not taken antihypertensive medication in the Korean National Health Service-National Health Screening Cohort between 2002 and 2003. Participants were categorized into four groups according to 2017 American College of Cardiology/American Heart Association hypertension guideline. Primary outcome was MACE characterized by cardiovascular mortality, myocardial infarction, unstable angina, and stroke. During a 10-year follow-up, a significant increase in MACE risk was observed from the stage 1 hypertension group (hazard ratio [HR], 1.23; 95% CI 1.15–1.32; *P* < 0.001) in time-varying Cox analysis. This relationship was persistent in subjects aged < 70 years, but increased MACE risk was observed only in the stage 2 hypertension group in ≥ 70 years (HR, 1.52; 95% CI 1.32–1.76, *P* < 0.001). When categorized as per sex, both men and women showed significant MACE risk from stage 1 hypertension. However, on comparing the sexes after stratifying by age, a significantly increased risk of MACE was shown from stage 1 hypertension in men aged < 50 years, but from stage 2 hypertension in men aged ≥ 50 years. Meanwhile, increased MACE risk was observed from stage 2 hypertension in women aged < 60 years, but from stage 1 hypertension in women aged ≥ 60 years. Thus, young male subjects had higher MACE risk than young female subjects, but this difference gradually decreased with age and there was no difference between sexes in subjects aged ≥ 70 years. Therefore, our results suggest that hypertension treatment initiation may need to be individualized depending on age and sex.

## Introduction

Over several decades, hypertension (HTN) has been defined as systolic blood pressure (SBP) ≥ 140 mmHg or diastolic blood pressure (DBP) ≥ 90 mmHg. However, several meta-analyses conducted based on large observational studies have continuously reported that pre-HTN stage is also associated with increased cardiovascular disease (CVD) risk^1–4^. Additionally, recent Systolic Blood Pressure Intervention Trial (SPRINT) showed that intensive BP control at < 120 mmHg resulted in significantly lower rates of CVD compared with standard BP control^5^. Based on this evidence, 2017 American College of Cardiology/American Heart Association (ACC/AHA) guideline suggested to lower the threshold and newly defined HTN as SBP ≥ 130 mmHg or DBP ≥ 80 mmHg^6^. The guideline recommended that antihypertensive medications should be initiated when patients with high risk of CVD had SBP ≥ 130 mmHg or DBP ≥ 80 mmHg. However, the 2018 European Society of Cardiology/European Society of Hypertension (ESC/ESH) guideline still follows the classic target of SBP < 140 mmHg and DBP < 90 mmHg because the abovementioned studies had only been conducted for the patients who had received antihypertensive medications^7^. Thus, clinicians are confused about determining the time at which antihypertensive treatment should be initiated for the patients who do not receive any antihypertensive medications^8,9^.

Moreover, although recent HTN guidelines provided age-specific treatment options^6,7^, they suggested different starting points for high BP management. The 2018 ESC/ESH guideline suggested initiation of antihypertensive treatment when elderly subjects ≥ 65-year-old had SBP ≥ 140 mmHg and DBP ≥ 90 mmHg and subjects ≥ 80-year-old had SBP ≥ 160 mmHg and DBP ≥ 90 mmHg, respectively^7^. Meanwhile, the 2017 ACC/AHA guideline suggested the same BP threshold and goal for the elderly as those for the young adult^10^. In addition, although there is sex-specific difference in HTN and associated CVD risk^11^, no sex-specific treatment option has been established in recent guidelines to date.

Accordingly, this study aimed to investigate the relationship between BP and CVD risk in a large Korean subjects who had not previously been treated with antihypertensive medications. Especially, CVD risk according to BP was evaluated in subgroups of age and sex.

## Methods

### Study subjects

Individuals who were aged ≥ 40 years and had undergone health screening by Korean Medical Insurance Corporation in a National Health Insurance Service-National Health Screening Cohort (NHIS-HEALS) between 2002 and 2003 were enrolled^12^. Among 514,339 individuals who received health screening during this period, we excluded the following individuals: (1) 67,003 individuals who had a history of CVD, stroke, DM, CKD, and end-stage renal disease (ESRD); (2) 1128 individuals who died between 2002 and 2003 or lost to follow-up; (3) 103,369 individuals who were prescribed antihypertensive medications at baseline; and (4) 15,511 subjects without additional measured BP during the follow-up period. Finally, a total of 327,328 individuals were analyzed as subjects of this study (Supplementary Fig. S1 online). The study protocol was approved by the Institutional Review Board of Ewha Womans University, College of Medicine, Republic of Korea (EUMC 2018-01-039). Informed consent was waived owing to the retrospective nature of this study.

### Data collection

The NHIS-HEALS cohort consists of health examination records including demographic and anthropometric data as well as laboratory test details. Health-related behaviors, such as smoking status and drinking habit, were evaluated using self-reported questionnaire. BP was measured at mobile examination centers by qualified examiners in the sitting position after making the subjects rest for at least 5 min. When SBP was > 120 mmHg or DBP was > 80 mmHg, it was re-measured after at least an interval of 2 min. Laboratory tests included measurements of total cholesterol and fasting glucose levels via blood sampling after > 8 h of fasting. The Charlson comorbidity index (CCI) was calculated by assessing medical records^13^.

### Classification of HTN

All subjects were categorized into four groups according to 2017 ACC/AHA guideline: normal (SBP, < 120 mmHg and DBP, < 80 mmHg), elevated (SBP, 120–129 mmHg and DBP, < 80 mmHg), stage 1 HTN (SBP, 130–139 mmHg or DBP, 80–89 mmHg), and stage 2 HTN (SBP, ≥ 140 mmHg or DBP, ≥ 90 mmHg). BP was measured and its stage was identified at each visit during the entire follow-up period.

### Outcome assessment

The primary outcome of the study was major adverse cardiovascular events (MACE), which was defined as cardiovascular mortality, hospitalization due to myocardial infarction, unstable angina, or stroke (International Classification of Disease, Tenth Revision codes I20-I25 or I63-I64). Additionally, hospitalization was defined as admission to a hospital for at least consecutive 2 days. The occurrence of MACE was monitored for 10 years from January 1, 2004 to December 31, 2013^14^.

### Statistical analysis

Continuous variables were expressed as arithmetic or geometric mean ± standard deviation, and categorical variables were summarized as numbers and percentages. Comparisons between groups were conducted using Student’s *t* test and Pearson’ chi-square test, as appropriate. The incidence of MACE was expressed as 1000 person-year during follow-up, and confidence intervals (CIs) were estimated by Poisson distribution. Multivariable Cox regression analysis was performed to compare MACE risk in each BP stage. Moreover, with regard to both time-varying BP stages and time-varying covariates, time-varying Cox model was used to evaluate the relationship between BP stages and MACE in each age and sex group. In the models, age (years; continuous), body mass index (BMI) (< 25, 25–29.9; ≥ 30 kg/m^2^; and missing data), total cholesterol level (< 200; 200–239; ≥ 240 mg/dL; and missing data), fasting blood glucose level (< 100 and 100–125 mg/dL), smoking status (none-, past-, current smoker, and missing data), and drinking habit (none; once/twice a week; three/four times a week; more than five times a week; and missing data) at each visit were included as time-varying covariates, and sex (male and female) and CCI at baseline were included as fixed covariates. Right-censoring occurred due to administrative censoring, non-cardiovascular deaths, loss to follow-up, or use of antihypertensive medications during the follow-up period. Moreover, the marginal effects of sex were estimated in the association between BP components (SBP and DBP) and MACE to compare the effects in male and female. Moreover, the marginal effects of sex in the association between BP components (SBP and DBP) and MACE were estimated based on multiplicative interaction between each BP component and sex group^15^. A *p* value of < 0.05 was considered statistically significant. All statistical analyses were performed using R software version 4.0.1^16^ and SAS version 9.4 (SAS Institute Inc, Cary, NC, USA).

## Results

### Baseline characteristics

Baseline characteristics of subjects are presented in Table 1. When we classified the participants based on their baseline BP, 99,290 (30.3%) individuals were in the normal BP group and 30,745 (9.4%) were in the elevated BP group; furthermore, 111,240 (34.0%) had stage 1 HTN and 86,053 (26.3%) had stage 2 HTN. BP tends to be higher among older subjects, male sex, heavy alcohol drinkers, current smokers, and those who have higher serum total cholesterol and fasting blood glucose levels. However, CCI was lower in the higher BP group compared with that in the other groups.

**Table 1:** Baseline characteristics according to BP classification categorized by 2017 ACC/AHA Hypertension Guideline.

Variables	Total	BP classification				*P* value
		Normal BP	Elevated BP	Stage 1 HTN	Stage 2 HTN	
Total	327,328 (100)	99,290 (30.3)	30,745 (9.4)	111,240 (34.0)	86,053 (26.3)	
**Age group**						< 0.001
40–49	173,444 (53.0)	60,055 (60.5)	16,275 (53.0)	59,912 (53.9)	37,202 (43.2)	
50–59	92,797 (28.4)	25,824 (26.0)	8434 (27.4)	31,601 (28.4)	26,938 (31.3)	
60–69	48,853 (14.9)	11,020 (11.1)	4780 (15.6)	16,025 (14.4)	17,028 (19.8)	
≥ 70	12,234 (3.7)	2391 (2.4)	1256 (4.1)	3702 (3.3)	4885 (5.7)	
Male sex	180,766 (55.2)	42,497 (42.8)	15,589 (50.7)	66,373 (59.7)	56,307 (65.4)	< 0.001
**Body mass index (kg/m**^**2**^**)**						0.293
< 25.0	225,730 (69.0)	77,830 (78.4)	21,885 (71.2)	74,997 (67.4)	51,018 (59.3)	
25.0–29.9	94,860 (29.0)	20,477 (20.6)	8361 (27.2)	33,957 (30.5)	32,065 (37.3)	
≥ 30.0	6526 (2.0)	914 (0.9)	480 (1.6)	2209 (2.0)	2923 (3.4)	
Missing	212 (0.1)	69 (0.1)	19 (0.1)	77 (0.1)	47 (0.1)	
SBP (SD) (mmHg)	123.48 (16.3)	106.39 (7.3)	121.96 (2.8)	124.45 (7.7)	142.49 (12.9)	< 0.001
DBP (SD) (mmHg)	77.85 (11.0)	66.72 (6.0)	70.90 (4.5)	79.96 (3.9)	90.44 (8.2)	< 0.001
**Total cholesterol (mg/dL)**						< 0.001
< 200	176,435 (53.9)	59,001 (59.4)	16,967 (55.2)	58,839 (52.9)	41,628 (48.4)	
200–239	108,238 (33.1)	30,039 (30.3)	9930 (32.3)	37,618 (33.8)	30,651 (35.6)	
≥ 240	42,371 (12.9)	10,157 (10.2)	3820 (12.4)	14,695 (13.2)	13,699 (15.9)	
Missing	284 (0.1)	93 (0.1)	28 (0.1)	88 (0.1)	75 (0.1)	
**Fasting blood glucose (mg/dL)**						0.008
< 100	250,513 (76.5)	81,297 (81.9)	23,445 (76.3)	84,579 (76.0)	61,192 (71.1)	
100–125	76,815 (23.5)	17,993 (18.1)	7300 (23.7)	26,661 (24.0)	24,861 (28.9)	
**Smoking**						< 0.001
Non-smoker	206,617 (63.1)	68,676 (69.2)	20,117 (65.4)	67,950 (61.1)	49,874 (58.0)	
Past smoker	28,058 (8.6)	6751 (6.8)	2554 (8.3)	10,210 (9.2)	8543 (9.9)	
Current smoker	79,487 (24.3)	19,986 (20.1)	6916 (22.5)	28,608 (25.7)	23,977 (27.9)	
Missing	13,166 (4.0)	3877 (3.9)	1158 (3.8)	4472 (4.0)	3659 (4.3)	
**Drinking habit**						< 0.001
None	227,861 (69.6)	76,686 (77.2)	22,447 (73.0)	75,551 (67.9)	53,177 (61.8)	
Once or twice a week	56,937 (17.4)	13,594 (13.7)	4820 (15.7)	20,660 (18.6)	17,863 (20.8)	
Three or four times a week	23,173 (7.1)	4593 (4.6)	1812 (5.9)	8410 (7.6)	8358 (9.7)	
More than five times a week	13,582 (4.2)	2525 (2.5)	1105 (3.6)	4729 (4.3)	5223 (6.1)	
Missing	5775 (1.8)	1892 (1.9)	561 (1.8)	1890 (1.7)	1432 (1.7)	
Charlson comorbidity index (SD)	0.32 (0.6)	0.34 (0.7)	0.33 (0.6)	0.32 (0.6)	0.29 (0.6)	< 0.001

Data are expressed as mean ± standard deviation or No. (%).

*ACC/AHA* American College of Cardiology and American Heart Association, *SBP* systolic blood pressure, *DBP* diastolic blood pressure, *HTN* hypertension.

### Risk of MACE in each BP stage

During a 10-year monitoring, the median follow-up duration was 8.61 years and 53.2% of subjects were censored during follow-up period due to start of antihypertensive medication (Supplementary Table S1 online). The overall incidence rate of MACE was 2.41 per 1000 person-years. The crude incidence rate (CIR) for MACE in the normal BP group was 1.67 per 1000 person-years, which gradually increased with an increase in BP stage (Table 2). Kaplan–Meier curves reflected a significant cumulative increase in the incidence rate of MACE in each of the elevated BP groups, i.e., stage 1 and 2 HTN groups, compared with the incidence rate of MACE in the normal BP group (Supplementary Fig. S2 online). However, according to the results of multivariable Cox regression analysis, the risk of MACE was not significantly increased in the elevated BP group but was significantly higher in the stage 1 HTN group [hazard ratio (HR), 1.24; 95% CI 1.16–1.33; *P* < 0.001] and stage 2 HTN group (HR, 1.60; 95% CI 1.48–1.72; *P* < 0.001) than that in the normal BP group. The time-varying cox model also showed consistent results with the conventional Cox proportional model (Table 2).

**Table 2:** Incidence rate of MACE and its comparisons in each BP classification.

Group	Number of subjects (N = 327,328)	Incidence rate			Cox proportional model			Time-varying cox model	
		Events per 1000 person-years	95% CI		Hazard ratio^a^	95% CI		Hazard ratio^a^	95% CI
Normal BP	99,290 (30.3)	1.67	1.59–1.76		Reference			Reference	
Elevate BP	30,745 (9.4)	2.10	1.92–2.29		1.08	0.97–1.19		1.07	0.97–1.18
Stage 1 HTN	111,240 (34.0)	2.45	2.35–2.56		1.24	1.16–1.33		1.23	1.15–1.32
Stage 2 HTN	86,053 (26.3)	3.66	3.50–3.83		1.60	1.48–1.72		1.66	1.54–1.79

*MACE* major adverse cardiovascular events, *HTN* hypertension, *BP* blood pressure, *CI* confidence interval, *BMI* body mass index, *CCI* Charlson comorbidity index.

^a^The model was used with adjusting age, sex, BMI, total cholesterol, fasting glucose, smoking status, drinking habit, CCI, and calendar year.

### Risk of MACE in each BP stage after stratified by age

The proportion of patients censored due to start of antihypertensive medication tended to less in the younger groups (Supplementary Table S1 online). The CIRs (events per 1000 person-years) of MACE gradually increased with increasing age. For subjects aged 70 years, the incidence rate of MACE in the normal BP group was 9.22 (7.73–10.91), whereas it was 8.77 (6.72–11.26) in the elevated BP group (Supplementary Table S2 online). Cox regression analysis revealed a significant interaction of age on the relationship between BP and risk of MACE (*P* for interaction < 0.001). Multivariate time-varying Cox regression analysis showed significantly increased risk of MACE in stage 1 and 2 HTN groups from the age of 40 years to 60 years, whereas, in elderly subjects aged ≥ 70 s, the risk of MACE was significantly increased only in the stage 2 HTN group (HR, 1.56; 95% CI 1.25–1.95; *P* < 0.001) (Table 3).

**Table 3:** Comparisons of the incidence rate of MACE in each BP classification stratified based on each age and sex.

Group	Age groups				Sex	
	40–49	50–59	60–69	70+	Male	Female
No. of subjects	173,444	92,797	48,853	12,234	180,766	146,562
Normal BP	Reference	Reference	Reference	Reference	Reference	Reference
Elevate BP	1.14 (0.97–1.35)	1.12 (0.95–1.35)	1.01 (0.83–1.23)	0.88 (0.62–1.22)	1.04 (0.92–1.17)	1.12 (0.95–1.32)
Stage 1 HTN	1.34 (1.20–1.50)	1.18 (1.05–1.34)	1.28 (1.12–1.47)	1.22 (0.98–1.52)	1.19 (1.10–1.30)	1.30 (1.16–1.46)
Stage 2 HTN	2.03 (1.78–2.31)	1.71 (1.49–1.96)	1.52 (1.32–1.76)	1.56 (1.25–1.95)	1.70 (1.56–1.86)	1.51 (1.31–1.73)

The models was conducted with time-varying Cox analysis after adjusting age, sex, BMI, total cholesterol, fasting glucose, smoking status, drinking habit, CCI and calendar year.

*MACE* major adverse cardiovascular events, *HTN* hypertension, *BP* blood pressure, *CI* confidence interval, *BMI* body mass index, *CCI* Charlson comorbidity index, *No.* number.

### Risk of MACE in each BP stage after stratified by sex

When the study subjects were classified according to their sex, CIR of MACE in males [3.01 (2.92–3.10)] was higher than that in females [1.65 (1.58–1.73)] (Supplementary Table S3 online). There was also a significant interaction of sex on the association between BP classification and incidence rate of MACE (*P* for interaction = 0.04). However, the risk of MACE significantly increased in the stage 1 and 2 HTN groups compared with that in the normal BP group, irrespectively of sex (Table 3).

### Risk of MACE in each BP stage after stratified by both age and sex

Therefore, we stratified these subjects based on both age and sex. Among male subjects aged 40 s, both stage 1 and 2 HTN groups (SBP ≥ 130 mmHg or DBP ≥ 80 mmHg) showed a significant increase risk of MACE compared with the normal group (Table 4). However, in male subjects aged ≥ 50 years, significantly increased risk of MACE was only shown in stage 2 HTN groups (SBP ≥ 140 mmHg or DBP ≥ 90 mmHg). Meanwhile in female subjects, only stage 2 HTN group showed a significant increase risk of MACE in the group aged < 60 years. However, risk of MACE in stage 1 HTN became prominent in older female subjects, and MACE risk was significantly increased from stage 1 HTN in female subjects aged ≥ 60 years.

**Table 4:** Time varying cox analysis of hypertension stages for major adverse cardiovascular events according to both age and sex groups.

Group	40–49		50–59		60–69		70+	
	HR	95% CI	HR	95% CI	HR	95% CI	HR	95% CI
**Male**								
Normal BP	Reference		Reference		Reference		Reference	
Elevate BP	1.13	(0.93–1.38)	1.02	(0.82–1.28)	1.02	(0.79–1.3)	0.68	(0.42–1.11)
Stage 1 HTN	1.30	(1.14–1.48)	1.11	(0.95–1.29)	1.19	(1.00–1.41)	1.00	(0.74–1.34)
Stage 2 HTN	1.97	(1.70–2.29)	1.74	(1.48–2.05)	1.39	(1.15–1.66)	1.50	(1.12–2.00)
**Female**								
Normal BP	Reference		Reference		Reference		Reference	
Elevate BP	1.09	(0.79–1.49)	1.30	(0.98–1.73)	0.98	(0.71–1.35)	1.14	(0.70–1.87)
Stage 1 HTN	1.20	(0.96–1.50)	1.21	(0.98–1.51)	1.37	(1.10–1.71)	1.54	(1.10–2.16)
Stage 2 HTN	1.59	(1.18–2.15)	1.25	(0.95–1.64)	1.65	(1.31–2.09)	1.63	(1.16–2.31)

The models was used with adjusting age, BMI, total cholesterol, fasting glucose, smoking status, drinking habit, CCI and calendar year.

*HTN* hypertension, *BP* blood pressure, *BMI* body mass index, *CCI* Charlson comorbidity index, *HR* hazard ratio, *CI* confidence interval.

**Figure 1:**
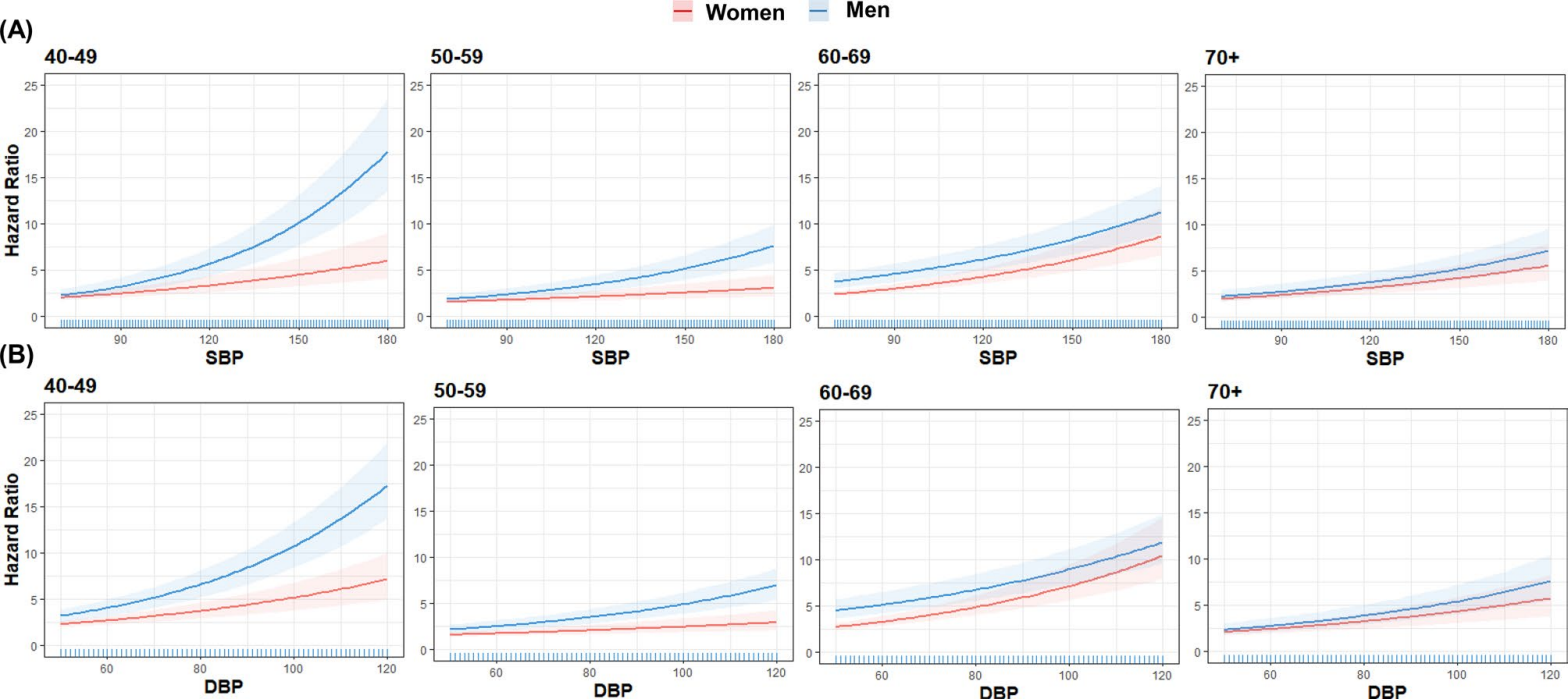
Hazard ratio for cardiovascular disease event and stroke according to each blood pressure component. Hazard ratios were calculated by time-varying Cox regression analysis after adjusting for age, sex, body mass index, total cholesterol level, fasting glucose level, smoking status, alcohol intake, and Charlson Comorbidity Index score. The solid line represents the estimate; the shaded area represents uncertainty bounds among estimates calculated via 10,000 simulations. Figure was generated using R software version 4.0.1^16^.

**Figure 2:**
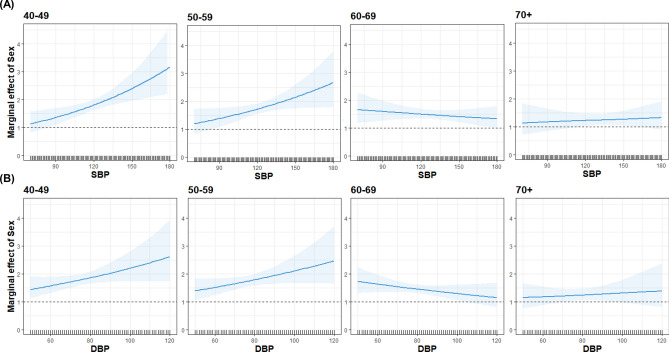
Marginal effect of sex (men relative to women) at specific values of (**A**) systolic blood pressure and (**B**) diastolic blood pressure. Marginal effects were calculated based on the interaction between blood pressure components and sex group in the time-varying cox model after making adjustments for age, sex, body mass index, total cholesterol level, fasting glucose level, smoking status, alcohol intake, and Charlson Comorbidity Index score. The solid line represents the estimate; the shaded area represents uncertainty bounds among estimates calculated via 10,000 simulations. Figure was generated using R software version 4.0.1^16^.

Figure 1 shows a head-to-head comparison of each BP component (SBP and DBP) with MACE risk between sexes. The nonlinear curves of both SBP and DBP showed steeper HR curves with increasing BP for males than for females. However, the discrepancies between sexes with regard to the curves of both SBP and DBP gradually reduced with increasing age. Moreover, a marginal effect plot revealed that males had a higher risk of MACE than females, even they had the same BP, especially in a younger subjects. However, the slope of curves became gentle with increasing age, and there was no significant difference in MACE risk between sexes in groups comprising ≥ 70-year-old subjects (Fig. 2).

Furthermore, we investigated whether risk of MACE would differ based on the cause of MACE [coronary heart disease (CHD) and stroke]. Only the subjects aged between 40 and 60 years belonging to the stage 2 HTN group had a higher risk of CHD than those belonging to the normal BP group, whereas the occurrence rate of stroke in all age groups was significantly associated with stage 1 and 2 HTN (Supplementary Table S4 online). Additionally, males with stage 1 and 2 HTN had a higher risk of CHD than those in the normal BP group, whereas no significant association was found among females (Supplementary Table S5 online). However, the stage 1 and 2 HTN groups showed a significant relationship with an increased risk of stroke, irrespectively of sex.

## Discussion

Here, we demonstrated the natural course of MACE development in association with BP according to age and sex of the subjects who did not receive any antihypertensive medication. Subjects with HTN, classified according to 2017 ACC/AHA guideline as either stage 1 and 2 HTN, had a significantly higher risk of MACE than those with normal BP. However, this relationship was not persistent among all age groups and sexes. Younger subjects had a greater risk of MACE from stage 1 HTN, whereas elderly subjects aged ≥ 70 years only belonging to the stage 2 HTN group showed a significant association with MACE occurrence. Moreover, age interaction was not shown similarly in both sexes. A significantly increased risk of MACE in stage 1 HTN was shown in male subjects younger than 50 years old, whereas in female subjects, it was only shown aged ≥ 60 years. Therefore, we found that the male subjects had higher MACE risk than the female subjects in younger age groups, but the differences in MACE risk between sexes gradually reduced in the elderly age group.

When subjects were stratified by age, younger subjects tended to have a higher risk of MACE than older subjects even when they had similar BPs. In a recent study conducted in the United States on young adults aged < 40 years, even the individuals in elevated BP group had a significantly higher risk of subsequent CVD events than those with normal BP^17^. Moreover, a large-scale study conducted in South Korea, which included more than 2 million young adults aged < 40 years, also reported similar results of a significantly higher CVD risk among individuals with elevated BP group^18^. One may think that these results were caused by low awareness and low treatment rate of HTN in young adults^19,20^. However, because we excluded subjects of all age groups who had been treated with antihypertensive medications, it can be suggested that young adults are more vulnerable to even a small increase in BP. Accordingly, early initiation of HTN treatment will need to be applied more strictly in young adults.

Contrastingly, in our study, MACE risk was decreased with increasing age when compared with normal BP in each age group, and subjects aged ≥ 70 years had significantly increased MACE risk from stage 2 HTN. Previously, Rapsomaniki et al. reported an age-specific association between BP and twelve CVDs in a cohort of 1.25 million patients^21^. In that study, increasing BP had a strong association with CVDs across a wide age range, but these associations were reduced with increasing age. However, several randomized controlled trials (RCTs) produced inconsistent results regarding the relationship between BP and CVD in the elderly. Two RCTs conducted in Japan reported no benefit for an SBP goal of < 140 mmHg in the elderly^22,23^. Meanwhile, *post-hoc* analysis of SPRINT, which was conducted in patients aged ≥ 75 years, revealed that an intensive BP management at < 120 mmHg resulted in lower rates of CVD^10^. Thus, the 2017 ACC/AHA guideline did not recommend different BP thresholds for the elderly. However, RCTs were usually conducted for patients with high CVD risk and those who were already receiving antihypertensive medications. We included subjects with a relatively low risk of CVD after excluding those with a previous history of CVD, DM, or CKD as well as those who had already taken antihypertensive medications. Thus, application of the 2017 ACC/AHA guideline may be too aggressive for the elderly at low CVD risk. The HOPE-3 trial, which was conducted on subjects with intermediate CVD risk of whom only 22% had a history of antihypertensive treatment, did not show a reduction in CVD risk after BP-lowering treatment^24^. Thus, it may be possible to delay the initiation of antihypertensive treatment until BP reaches stage 2 HTN (SBP ≥ 140 mmHg or DBP ≥ 90 mmHg) in low-risk elderly.

Furthermore, on evaluating the association between BP category and MACE risk depending on sex, both men and women were found to have a significantly increased risk of MACE from stage 1 HTN. However, when we classified subjects by both age and sex, MACE risk did not appear to be similar between the sexes of each age group. It is well known that premenopausal women have lower BP than men of similar age group. However, BP increases sharply after menopause in women, and eventually, the prevalence of HTN in women surpasses that in men with increasing age^11^. In Korea, the prevalence of HTN was higher in men until the age of 60 years, but it became higher in women from the age of 70 years^20^. This disparity between sexes can be partially explained by lifestyle factors such as smoking and alcohol consumption. However, inherited factors such as sex hormones and sex chromosome complement can affect BP and CVD development by modulating the sympathetic nervous system, renin–angiotensin–aldosterone system, and immune system^11^. These regulatory processes in women are balanced toward cardio-protection during the reproductive period, but they worsened after menopause. Our results clearly showed that male subjects were at a greater risk of MACE than female subjects, and this difference between sexes tended to be greater at a younger age. Our results also can be supported by a recent *post-hoc* analysis of SPRINT^25^. After sex-specific propensity score-matched analysis between intensive and standard BP control, the aforementioned study reported that intensive BP control was effective in lowering CVD risk in men (HR, 0.70; 95% CI 0.57–0.86) but not in women (HR, 0.82; 95% CI 0.60–1.12). The results of that study pertaining to women benefiting lesser from intensive therapy can be explained by our results that women had lower MACE risk than men. However, as aforementioned, participants of SPRINT had different characteristics from ours. Especially, a large proportion of SPRINT participants were elderly. Because our marginal effect plot revealed a significantly higher MACE risk among young male subjects than the young female subjects even when both sexes had the same BP, it can be suggested that BP threshold for medication initiation can be lowered for young adult men than that for young adult women.

This study had several limitations. First, although we used time-varying BP to avoid potential errors of BP measurements, we used only office BP and there were possibilities for unidentified white coat and masked hypertension. Some observational studies and meta-analysis reported the superiority of ambulatory BP than the conventional office BP for CVD and mortality prediction^26,27^. Therefore, our results may be confounded by BP measurement per se. Second, because we conducted analyses for subjects who did not take antihypertensive medications, our findings can be an evidence for the initiation of a new antihypertensive treatment but cannot be the basis of setting BP goal for patients who are already taking antihypertensive medications. A recent observational study reported that the relationship between BP and CVD outcome could vary according to the use of antihypertensive medications^28^. However, regarding the initiation of a new antihypertensive treatment, RCTs may give rise to ethical issues pertaining to the delay in antihypertensive treatment for hypertensive patients of the placebo arm. Therefore, large-scale observational studies and meta-analyses may be the best evidences for this issue. Finally, this study was conducted for the Korean subjects; hence, our findings cannot be generalized to individuals from other countries with different socioeconomic status and race. Despite these limitations, the study was conducted by including a large subjects and attempts were made to meet individual requirements for BP management after stratification by age and/or sex.

In conclusion, HTN, which was defined by the 2017 ACC/AHA guideline, was associated with an increased risk of MACE in the Korean who had low CVD risk and did not receive antihypertensive medication. However, a high risk of MACE caused by increasing BP was prominent at a younger age, and it tended to reduce as the subjects got older. Furthermore, men had a significantly higher risk of MACE than women, but a difference in MACE risk between sexes was only noticeable at younger ages. Accordingly, HTN treatment initiation needs to be done on an individual basis depending on age and sex.

## Supplementary Information

Supplementary Information.

## Data Availability

The NHIS of Korea, the data provider, requires all involved researchers to pledge not to share, release, or review the data with other entities. The researchers can access to the NHIS data directly via following URLs: https://nhiss.nhis.or.kr/bd/ab/bdaba000eng.do.
