# Supplementary material for: Age and sex specific target of blood pressure for the prevention of cardiovascular event among the treatment naive hypertensive patients
Source: Sci Rep. 2020 Dec 9;10:21538. doi: 10.1038/s41598-020-78641-3 (PMC7726552; doi:10.1038/s41598-020-78641-3)
Supplement: Supplementary file 1 — Supplementary Information. [file 41598_2020_78641_MOESM1_ESM.docx]

**Age and sex specific target of blood pressure for the prevention of cardiovascular event among the treatment naive hypertensive patients**

Hyoungnae Kim, MD, Seulbi Lee, PhD, Eunhee, Ha, MD, PhD, Soon Hyo Kwon, MD, PhD, Jin Seok Jeon, MD, PhD, Hyunjin Noh, MD, PhD, Dong Cheol Han, MD, PhD, Hyung Jung Oh, MD, PhD, and Dong-Ryeol Ryu, MD, PhD

**Online Supplementary Materials**

**Supplementary Table S1.** Number and follow-up duration of subjects according to start of antihypertensive medications

**Supplementary Table S2.** Number of subjects and observations, and incidence rate in the time-varying cox model of hypertension stages for major adverse cardiovascular events according to age groups

**Supplementary Table S3.** Number of subjects and observations, and incidence rate in the time-varying cox model of hypertension stages for major adverse cardiovascular events according to sex

**Supplementary Table S4.** Cause-specific time varying Cox analysis of hypertension stages for major adverse cardiovascular events according to age groups

**Supplementary Table S5.** Cause-specific time varying Cox analysis of hypertension stages for major adverse cardiovascular events according to sex groups

**Supplementary Fig. S1.** Flow diagram for participant selection

**Supplementary Fig. S2.** The cumulative incidence rate of major adverse cardiovascular events according to the 2017 ACC/AHA guideline for hypertension.

**Supplementary Table S1. Number and follow-up duration of subjects according to start of antihypertensive medications**

|  | **Total** | **Age groups** | | | | **Sex groups** | |
| --- | --- | --- | --- | --- | --- | --- | --- |
|  |  | **40-49** | **50-59** | **60-69** | **70+** | **Male** | **Female** |
| No. of subjects |  |  |  |  |  |  |  |
| Total (n) | 327,328 | 173,444 | 92,797 | 48,853 | 12,234 | 180,766 | 146,562 |
| Censored due to start of antihypertensives (n, %) | 174,187 (53.2) | 74,714 (43.1) | 55,053 (59.3) | 35,230 (72.1) | 9,190 (75.1) | 91,660 (50.7) | 82,527 (56.3) |
| Median follow-up duration (years) |  |  |  |  |  |  |  |
| Total | 8.61 | 9.20 | 7.45 | 5.25 | 4.18 | 8.99 | 8.22 |
| Censored due to start of antihypertensives | 4.60 | 5.12 | 4.37 | 3.79 | 3.26 | 4.89 | 4.77 |

**Supplementary Table S2. Number of subjects and observations, and incidence rate in the time-varying cox model of hypertension stages for major adverse cardiovascular events according to age groups**

| **Group** | **40-49** | | | | **50-59** | | | | **60-69** | | | | **70+** | | |
| --- | --- | --- | --- | --- | --- | --- | --- | --- | --- | --- | --- | --- | --- | --- | --- |
|  | **No. of Subject** | **No. of Obs.** | **Incidence rate*** | **No. of Subject** | | **No. of Obs.** | **Incidence rate*** | **No. of Subject** | | **No. of Obs.** | **Incidence rate^*^** | **No. of Subject** | | **No. of Obs.** | **Incidence rate^*^** |
| Total | 173,444 | 850,161 | 1.39  (1.33-1.46) | 92,797 | | 358,439 | 2.60  (2.48-2.72) | 48,853 | | 142,092 | 5.40  (5.13-5.68) | 12,234 | | 29,741 | 10.36  (9.57-11.19) |
| Normal BP | 60,055 | 313614 | 0.98  (0.99-1.07) | 25,824 | | 109409 | 2.03  (1.84-2.23) | 11,020 | | 35259 | 4.22  (3.77-4.71) | 2,391 | | 6,390 | 9.22  (7.73-10.91) |
| Elevated BP | 16,275 | 89754 | 1.17  (1.00 – 1.36) | 8,434 | | 38643 | 2.06  (1.73-2.44) | 4,780 | | 15596 | 5.04  (4.29-5.89) | 1,256 | | 3,101 | 8.77  (6.72-11.26) |
| Stage 1 HTN | 59,912 | 317198 | 1.51  (1.40-1.62) | 31,601 | | 133464 | 2.61  (2.41-2.83) | 16,025 | | 50910 | 5.47  (5.01-5.95) | 3,702 | | 10,022 | 10.19  (8.83-11.70) |
| Stage 2 HTN | 37,202 | 129595 | 2.15  (1.97-2.33) | 26,938 | | 76923 | 3.55  (3.26-3.86) | 17,028 | | 40327 | 6.52  (5.98-7.09) | 4,885 | | 10,228 | 11.75  (10.36-13.27) |

^*^ Events per 1,000 person-years

Abbreviations; No., number; Obs, observations;

**Supplementary Table S3. Number of subjects and observations, and incidence rate in the time-varying cox model of hypertension stages for major adverse cardiovascular events according to sex**

| **Group** | **Male** | | | **Female** | | |
| --- | --- | --- | --- | --- | --- | --- |
|  | **No. of Subject** | **No. of Obs** | **Incidence rate^*^** | **No. of Subject** | **No. of Obs** | **Incidence rate^*^** |
| Total | 180,766 | 827,735 | 3.01 (2.92-3.10) | 146,562 | 552,698 | 1.65 (1.58-1.73) |
| Normal BP | 42,497 | 228,675 | 2.27 (2.12-2.43) | 56,793 | 235,997 | 1.21 (1.11-1.31) |
| Elevated BP | 15,589 | 83,195 | 2.54 (2.27-2.83) | 15,156 | 63,899 | 1.62 (1.39-1.86) |
| Stage 1 HTN | 66,373 | 337,472 | 2.89 (2.74-3.04) | 44,867 | 174,122 | 1.76 (1.62-1.91) |
| Stage 2 HTN | 56,307 | 178,393 | 4.09 (3.88-4.31) | 29,746 | 78,680 | 2.73 (2.48-2.99) |

^*^ Events per 1,000 person-years

Abbreviations; No., number; Obs, observations;

**Supplementary Table S4. Cause-specific time varying Cox analysis of hypertension stages for major adverse cardiovascular events according to age groups**

| **Group** | **Normal BP** | | **Elevated BP** | | **Stage 1 HTN** | | **Stage 2 HTN** | |
| --- | --- | --- | --- | --- | --- | --- | --- | --- |
|  | **Estimate** | **95% CI** | **Estimate** | **95% CI** | **Estimate** | **95% CI** | **Estimate** | **95% CI** |
| **Age group (40-49)** |  |  |  |  |  |  |  |  |
| Coronary heart disease |  |  |  |  |  |  |  |  |
| Incidence^*^ | 0.12 | (0.09-0.15) | 0.12 | (0.07- 0.19) | 0.13 | (0.10- 0.16) | 0.22 | (0.17- 0.28) |
| Adjusted HR (95% CI)^†^ | 1 | (Reference) | 1.08 | (0.88-1.33) | 1.12 | (0.97-1.28) | **1.44** | **(1.21-1.70)** |
| Stroke |  |  |  |  |  |  |  |  |
| Incidence^*^ | 0.006 | (0.04-0.08) | 0.10 | (0.06-0.17) | 0.09 | (0.06- 0.11) | 0.18 | (0.13- 0.24) |
| Adjusted HR (95% CI) ^†^ | 1 | (Reference) | 1.17 | (0.86-1.59) | **1.57** | **(1.28-1.92)** | **2.71** | **(2.17-3.39)** |
| **Age group (50-59)** |  |  |  |  |  |  |  |  |
| Coronary heart disease |  |  |  |  |  |  |  |  |
| Incidence^*^ | 0.24 | (0.18- 0.31) | 0.19 | (0.10- 0.33) | 0.27 | (0.21- 0.35) | 0.34 | (0.25- 0.44) |
| Adjusted HR (95% CI) ^†^ | 1 | (Reference) | 1.03 | (0.81-1.30) | 1.06 | (0.89-1.25) | **1.20** | **(0.99-1.46)** |
| Stroke |  |  |  |  |  |  |  |  |
| Incidence^*^ | 0.14 | (0.10-0.20) | 0.18 | (0.10-0.31) | 0.22 | (0.17-0.29) | 0.38 | (0.29-0.49) |
| Adjusted HR (95% CI) ^†^ | 1 | (Reference) | 1.24 | (0.94-1.64) | **1.23** | **(1.01-1.51)** | **2.10** | **(1.70-2.60)** |
| **Age group (60-69)** |  |  |  |  |  |  |  |  |
| Coronary heart disease |  |  |  |  |  |  |  |  |
| Incidence^*^ | 0.47 | (0.33- 0.65) | 0.50 | (0.29- 0.80) | 0.66 | (0.51- 0.84) | 0.70 | (0.53- 0.90) |
| Adjusted HR (95% CI) ^†^ | 1 | (Reference) | 1.15 | (0.86-1.54) | 1.23 | (0.99-1.52) | **1.31** | **(1.04-1.65)** |
| Stroke |  |  |  |  |  |  |  |  |
| Incidence^*^ | 0.56 | (0.41- 0.76) | 0.56 | (0.34- 0.88) | 0.62 | (0.48-0.79) | 0.92 | (0.73- 1.14) |
| Adjusted HR (95% CI) ^†^ | 1 | (Reference) | 0.83 | (0.62-1.10) | **1.28** | **(1.06-1.54)** | **1.55** | **(1.27-1.89)** |
| **Age group (70+)** |  |  |  |  |  |  |  |  |
| Coronary heart disease |  |  |  |  |  |  |  |  |
| Incidence^*^ | 0.93 | (0.52-1.55) | 0.45 | (0.12- 1.24) | 1.21 | (0.78-1.78) | 0.99 | (0.63-1.48) |
| Adjusted HR (95% CI) ^†^ | 1 | (Reference) | 0.60 | (0.33-1.10) | 0.97 | (0.67-1.39) | 0.77 | (0.53-1.14) |
| Stroke |  |  |  |  |  |  |  |  |
| Incidence^*^ | 0.86 | (0.46-1.46) | 1.21 | (0.56- 2.30) | 0.89 | (0.54- 1.40) | 2.21 | (1.64 2.91) |
| Adjusted HR (95% CI) ^†^ | 1 | (Reference) | 1.22 | (0.77-1.92) | **1.56** | **(1.13-2.15)** | **2.42** | **(1.76-3.31)** |

^*^ Incidence were calculated by number of events / 1000 person-years.

^†^ Hazard ratio calculated by Time-varying Cox regression analysis after adjustments for sex, BMI, total cholesterol, fasting glucose, smoking status, drinking habit, CCI and calendar year.

Abbreviations; HTN, hypertension; BP, blood pressure; BMI, body mass index; CCI, Charlson comorbidity index; HR, Hazard ratio; CI, confidence interval.

**Supplementary Table S5. Cause-specific time varying Cox analysis of hypertension stages for major adverse cardiovascular events according to sex groups**

| **Group** | **Normal BP** | | **Elevated BP** | | **Stage 1 HTN** | | **Stage 2 HTN** | |
| --- | --- | --- | --- | --- | --- | --- | --- | --- |
|  | **Estimate** | **95% CI** | **Estimate** | **95% CI** | **Estimate** | **95% CI** | **Estimate** | **95% CI** |
| **Male** |  |  |  |  |  |  |  |  |
| Coronary heart disease |  |  |  |  |  |  |  |  |
| Incidence^*^ | 0.22 | (0.17- 0.27) | 0.24 | (0.16- 0.34) | 0.28 | (0.24-0.33) | 0.39 | (0.33- 0.46) |
| Adjusted HR (95% CI) ^†^ | 1 | (Reference) | 1.05 | (0.89-1.23) | **1.13** | **(1.01-1.26)** | **1.32** | **(1.16-1.50)** |
| Stroke |  |  |  |  |  |  |  |  |
| Incidence^*^ | 0.16 | (0.12- 0.20) | 0.22 | (0.15- 0.32) | 0.20 | (0.16- 0.24_ | 0.44 | (0.37- 0.52) |
| Adjusted HR (95% CI) ^†^ | 1 | (Reference) | 0.98 | (0.80-1.21) | **1.32** | **(1.15-1.51)** | **2.15** | **(1.87-2.48)** |
| **Female** |  |  |  |  |  |  |  |  |
| Coronary heart disease |  |  |  |  |  |  |  |  |
| Incidence^*^ | 0.18 | (0.14- 0.22) | 0.15 | (0.09- 0.24) | 0.21 | (0.16- 0.26) | 0.31 | (0.24- 0.41) |
| Adjusted HR (95% CI) ^†^ | 1 | (Reference) | 1.06 | (0.84-1.33) | 1.08 | (0.91-1.27) | 1.13 | (0.91-1.39) |
| Stroke |  |  |  |  |  |  |  |  |
| Incidence^*^ | 0.12 | (0.09- 0.16) | 0.19 | (0.12-0.29) | 0.21 | (0.16-0.26_ | 0.44 | (0.34-0.55) |
| Adjusted HR (95% CI) ^†^ | 1 | (Reference) | 1.25 | (0.98-1.59) | **1.51** | **(1.27-1.79)** | **1.85** | **(1.52-2.25)** |

^*^ Incidence were calculated by number of events / 1000 person-years.

^†^ Hazard ratio calculated by Time-varying Cox regression analysis after adjustments for age, BMI, total cholesterol, fasting glucose, smoking status, drinking habit, CCI and calendar year.

Abbreviations; HTN, hypertension; BP, blood pressure; BMI, body mass index; CCI, Charlson comorbidity index; HR, Hazard ratio; CI, confidence interval.


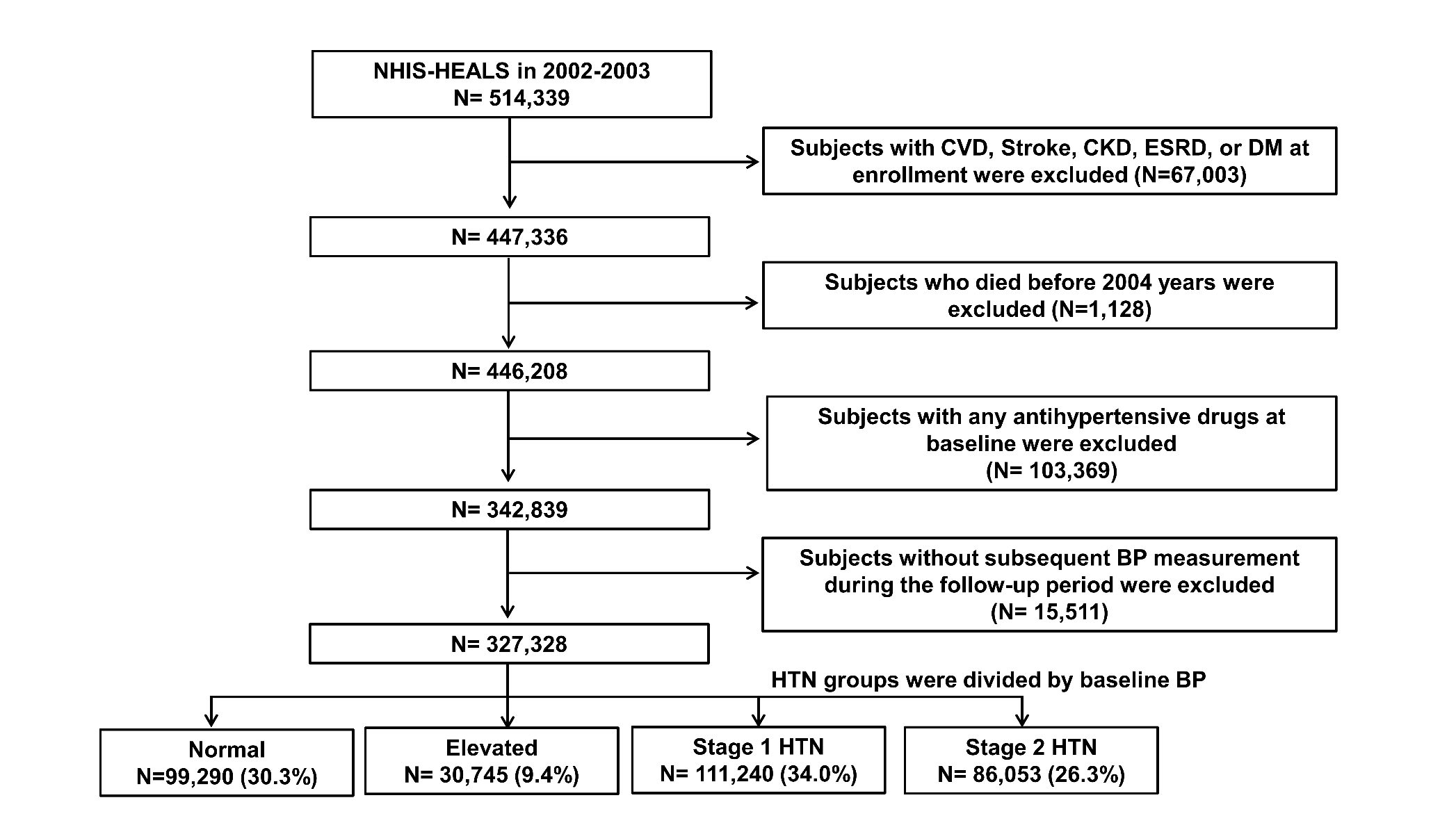


Supplementary Fig. S1. Flow diagram for participant selection


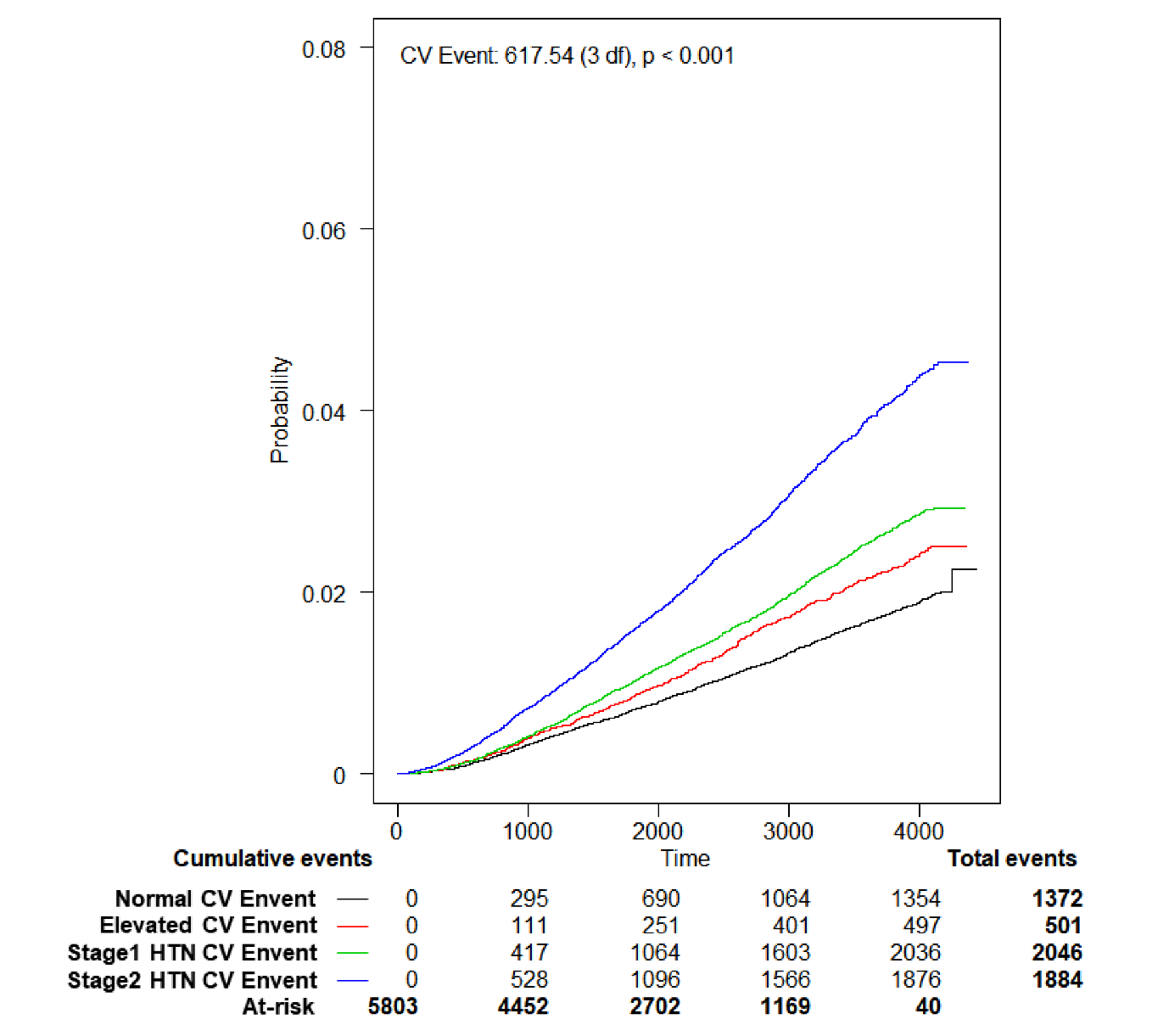


**Supplementary Fig. S2. The cumulative incidence rate of major adverse cardiovascular events according to the 2017 ACC/AHA guideline for hypertension.** Abbreviations: ACC/AHA, American college of cardiology/American Heart Association. Figure was generated using R software version 4.0.1. (R Core Team (2020). R: A language and environment for statistical computing. R Foundation for Statistical Computing, Vienna, Austria. http://www.R-project.org/).
